# Continuously tunable electronic structure of transition metal dichalcogenides superlattices

**DOI:** 10.1038/srep08356

**Published:** 2015-02-13

**Authors:** Yong-Hong Zhao, Feng Yang, Jian Wang, Hong Guo, Wei Ji

**Affiliations:** 1 College of Physics and Electronic Engineering, Institute of Solid State Physics, Sichuan Normal University, Chengdu 610068, China; 2 Department of Physics and the Center of Theoretical and Computational Physics, The University of Hong Kong, Hong Kong, China; 3 Department of Physics, Renmin University of China, Beijing 100872, China; 4 Beijing Key Laboratory of Optoelectronic Functional Materials & Micro-nano Devices, Renmin University of China, Beijing 100872, China; 5 Centre for the Physics of Materials and Department of Physics, McGill University, 3600 rue University, Montreal PQ, Canada H3A 2T8

## Abstract

Two dimensional transition metal dichalcogenides have very exciting properties for optoelectronic applications. In this work we theoretically investigate and predict that superlattices comprised of MoS_2_ and WSe_2_ multilayers possess continuously tunable electronic structure with direct bandgaps. The tunability is controlled by the thickness ratio of MoS_2_ versus WSe_2_ of the superlattice. When this ratio goes from 1:2 to 5:1, the dominant K-K direct bandgap is continuously tuned from 0.14 eV to 0.5 eV. The gap stays direct against −0.6% to 2% in-layer strain and up to −4.3% normal-layer compressive strain. The valance and conduction bands are spatially separated. These robust properties suggest that MoS_2_ and WSe_2_ multilayer superlattice should be a promising material for infrared optoelectronics.

It has been shown recently that monolayer (ML) transition metal dichalcogenides (TMDC) have very interesting properties as emerging materials for optoelectronic devices^1^,^2^,^3^,^4^,^5^,^6^,^7^. These ML-TMDCs have direct bandgaps in the visible frequency range and some of which have strong spin-orbit coupling (SOC). Prototypes of photoactive devices made by these materials already offer a quantum efficiency of up to 30%^8^ and a theoretical power conversion efficiency up to 1% in solar cell applications^9^. Interestingly, several TMDCs are indirect bandgap material in the bulk form, but it undergoes an indirect to direct transition when the thickness reduces to ML. These materials include MoS_2_, MoSe_2_, WS_2_, and WSe_2_^10^,^11^.

To realize a powerful TMDC optoelectronics, a most striking scenario is to make the bandgap tunable. While strain inside the layers of TMDC can linearly decrease the bandgap of monolayer MoS_2_^12^,^13^, however, it also makes the bandgaps of ML-TMDC indirect^14^. The bandgaps of hetero-bilayers, for instance a ML WS_2_ stacked on a ML WSe_2_, are predicted to be direct^15^,^16^. When the hetero-bilayers periodically repeat to form a superlattice, however, an *indirect* bandgap again emerges. It appears that ML-TMDC is paramount for retaining the direct bandgap^10^,^11^,^12^,^13^,^14^,^15^,^16^,^17^,^18^,^19^,^20^,^21^. This restriction strongly limits the fundamental building blocks for TMDC optoelectronics. In addition, a significant portion of solar spectrum is in the infrared range. A photovoltaic device that has narrower direct bandgap thus naturally absorbs more energy of sunlight. In light of these, a very important open problem is how to design TMDC systems having both tunable electronic structures and narrower direct bandgaps. It is the purpose of this work to provide a solution to this problem.

## Results

**Figure 1:**
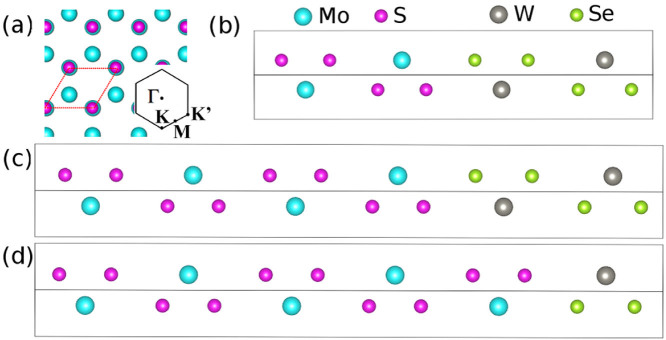
Crystal structure of representative superlattices. (a) top view of a superlattice as indicated by solid lines, and its associated first Brillouin zone; side views of (b) the 1:1 superlattice where MoS_2_:WSe_2_ equals to 1:1; (c) the 2:1 superlattice where MoS_2_:WSe_2_ equals to 2:1; (d) the 5:1 monolayer superlattice where all MoS_2_ and WSe_2_ bilayers were replaced by the corresponding monolayers.

In particular, we report a discovery that extends the candidates of building blocks for TMDC optoelectronic devices from only ML to potentially limitless possibilities. By first principles electronic structure theory we show that TMDC *multilayer* superlattices offer direct bandgap and continuously tunable electronic structures. The tunability is achieved by the thickness ratio of the multilayers across the hetero-interface. Namely, the 1:1 MoS_2_(bilayer)/WSe_2_(bilayer) superlattice, as shown in Fig. 1(a) and (b), has a direct bandgap of 0.33 eV which is continuously tunable by changing the MoS_2_ and WSe_2_ ratio. The direct bandgap is also robust against in-layer (*a* direction) strain from −0.6% to 2%, and normal-layer (*c* direction) uniaxial strain up to −4.3%. Moreover, the valance and conduction bands were found spatially separated. These interesting results strongly suggest that TMDC *multilayer* superlattices may well be the emerging optoelectronic device material in the mid-infrared frequency range.

Besides the 1:1 superlattice, two additional families of superlattices were investigated. In the first, the thickness of WSe_2_ is kept as constant, i.e. a bilayer, and the thickness of MoS_2_ is varied from bilayer to four layers (see Fig. 1 (c)) and eventually to ten layers. In other words, we increase the MoS_2_/WSe_2_ ratio (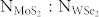) from 1:1 to 5:1. In the second family of superlattice, the thickness of MoS_2_ is kept fixed at bilayer and the ratio decreases from 1:1 to 1:5. Atomic structures including the volume and shape of the lattice and all internal atomic coordinates were fully optimized for each superlattice and the results were presented in Table 1. The associated in-layer lattice parameter *a* varies from 3.214 Å to 3.294 Å, close to the values of bulk MoS_2_ and WSe_2_ respectively, when the 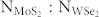 ratio decreases from 5:1 to 1:5.

**Figure 2:**
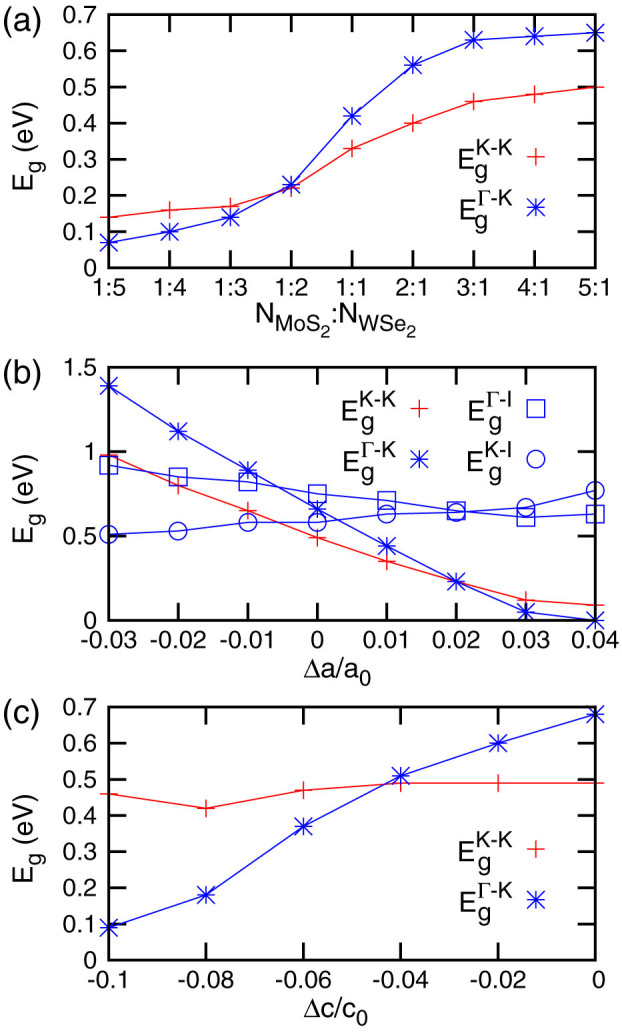
Direct (K-K) and indirect (Γ-K, Γ-I, and K-I) bandgaps of MoS_2_/WSe_2_ superlattice as a function of: (a) the MoS_2_ and WSe_2_ ratio; (b) the in-layer strain and (c) the normal-layer compressive strain. The red color and the plus symbols represent K-K direct gap; the blue color and the star, square and circle symbols represent the Γ-K, Γ-I and K-I indirect gaps, respectively.

Electronic properties of layered materials usually have a strong thickness dependence^22^,^23^. We firstly investigate the evolution of bandgaps as a function of the relative thickness of MoS_2_ and WSe_2_, i.e. the MoS_2_/WSe_2_ ratio. The strength of SOC is proportional to *Z*^4^ where *Z* is the effective atomic number. Transition metal atoms, like Mo and especially W, have a rather large atomic number, which shall give rise to a sufficiently large spin-orbit interaction and may qualitatively change the electronic structures around the Fermi Level. We, therefore, calculated the bandstructures with the inclusion of the SOC correction from the fully relaxed atomic structures of these superlattices. Figure 2(a) shows the values of the K-K direct and Γ-K indirect bandgaps as a function of the MoS_2_/WSe_2_ ratio. Both the direct and indirect gaps becomes larger as a function of this ratio. The largest direct (indirect) gap is 0.50 eV (0.65 eV) found at 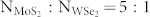; while the smallest direct (indirect) gap reaches 0.14 eV (0.07 eV) when 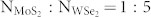. We conclude that both the direct and indirect bandgaps are continuously tunable, going from nearly zero-gap to narrow gap semiconductors according to the MoS_2_/WSe_2_ ratio of the superlattice. If all bilayers in the 5:1 superlattice are replaced with monolayers, as shown in Fig. 1(d), the superlattrice retains the direct bandgap of about 0.52 eV. The tuning of the bandgaps can be explained by the stacking and SOC induced VB separations. Very briefly, the thinner the WSe_2_ in the superlattice, the smaller the VB separation at the K point, hence the larger the K-K direct bandgap. In the monolayer case shown in Fig. 1(d), the stacking induced VB separation is further suppressed, giving rise to an even larger K-K gap. These behaviors will be elucidated further below, and we refer interested readers to Fig. S2(a) of the associated Supplementary Information for more details.

Strain significantly affects bandgaps of TMDC^6^,^14^,^24^,^25^ and usually changes the dominant bandgap of ML TMDC from direct to indirect as discussed above. While for multilayer WSe_2_, strain can induce a direct bandgap^26^. Our superlattice, however, resists such an undesirable change. As an example, we investigated the strain effect using the 5:1 superlattice which has the largest direct and indirect bandgaps among all superlattices we studied. Figures 2(b) and (c) plot the evolution of the bandgap as a function of in-layer and normal-layer strains, respectively. In-layer strain was applied by varying the in-layer lattice constant *a* and keeping it fixed during structural optimization, which ensures the structure relaxed with the optimized Poisson's ratio. Figure 2(b) plots four bandgaps, the K-K direct bandgap and the Γ-K, Γ-I, and K-I indirect bandgaps, versus Δ*a* from −3% to 4%. The K-K direct bandgap (red line) is dominant in these superlattices in a range of −0.6% ≤ Δ*a* ≤ 2.0%. Besides this range, the K-I or Γ-K indirect bandgaps are the smallest bandgaps. Normal-layer compressive strain was applied by the same scheme where the normal-layer lattice parameter *c* was shortened up to 10% at a step of 2%. Values of K-K direct and Γ-K indirect bandgaps were plotted in Fig. 2(c). The direct bandgap is almost a constant but the indirect gap rapidly decreases when the strain increases up to 10%, resulting in a transition from direct to indirect at a compressive ratio of ~−4.3%. This strain corresponds to an external pressure of ~3 GPa. We thus conclude that the proposed superlattices are robust against strain as far as the direct gap is concerned.

## Discussion

**Figure 3:**
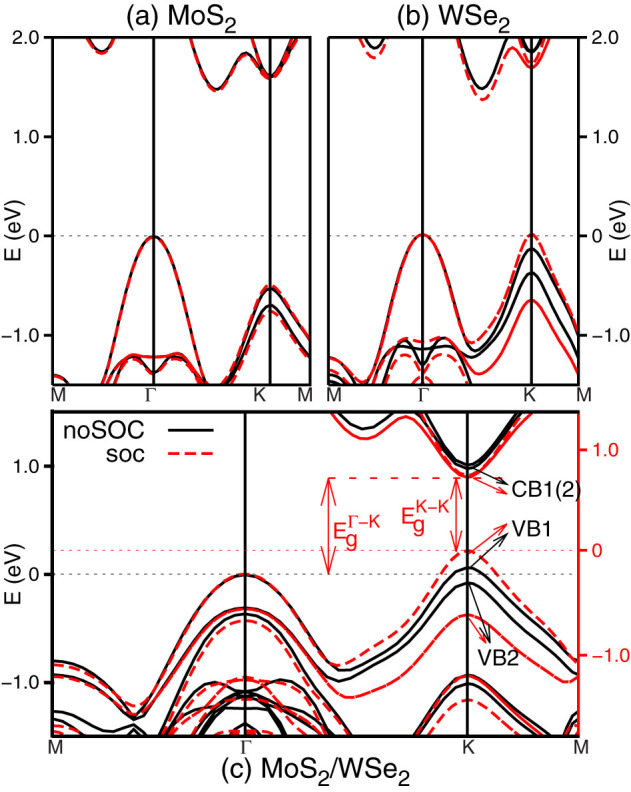
Band structures of (a) bulk MoS_2_ and (b) WSe_2_, and (c) the 1:1 superlattice shown in Fig. 1(a). Black solid and red dashed lines show the results without and with SOC, respectively. Horizontal thin dotted lines mark the energies of VBMs for non-SOC (black) and SOC (red) results. 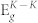 and 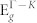 represent the direct and indirect gaps of the superlattice, respectively. States CB1, CB2, VB1, and VB2 are the four states around the bandgap.

**Figure 4:**
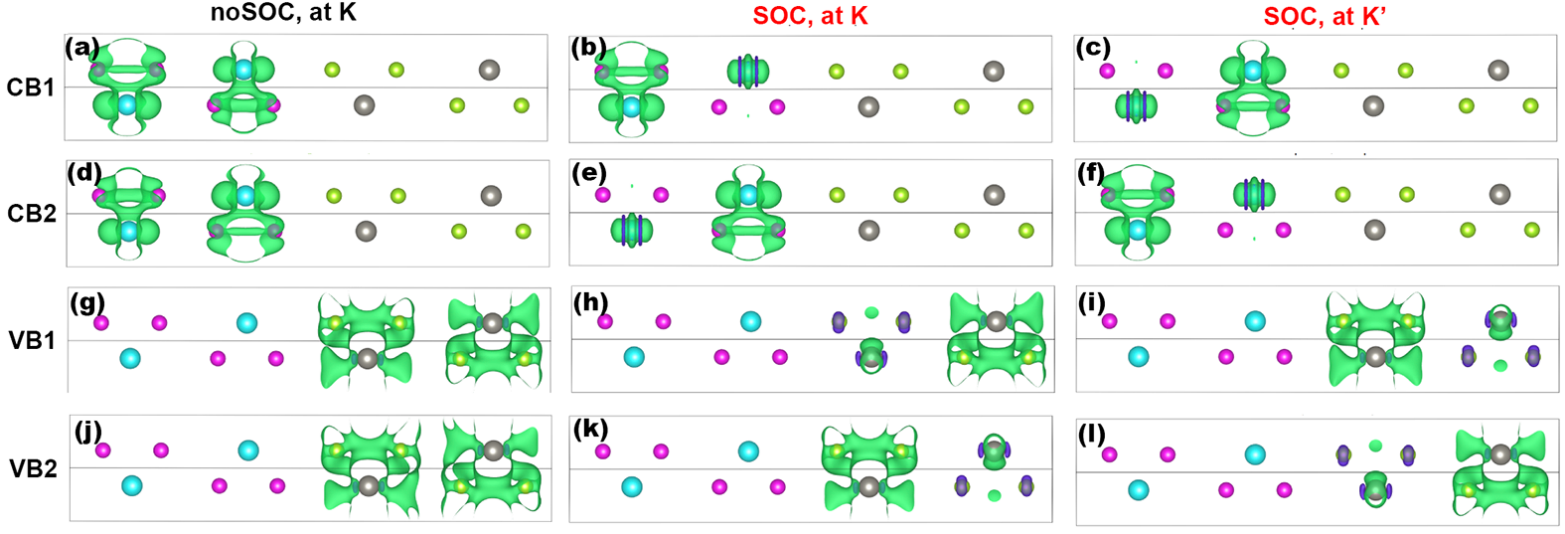
Visualized wavefunctions of the 1:1 superlattice, for CB1 (a)–(c), CB2 (d)–(f), VB1 (g)–(i) and VB2 (j)–(l), respectively. Non-SOC result at K and SOC results at K and K′ are shown at the left, middle and right panels, respectively. The isosurface is 0.001 *e*/Å^3^.

Having established the properties of tunable electronic structure and direct gap of the superlattices based on PBE-DFT calculation, we now discuss the origin of the direct bandgap by examining the bulk MoS_2_, WSe_2_ and the 1:1 superlattice, as shown in Fig. 3. Band structures based on the HSE06^27^ calculation are given in Fig. 3, in order to avoid the wellknown bandgap underestimation of PBE. Either with or without SOC, the bulk materials have indirect bandgaps between Γ and a certain intermediate point (I) along Γ-K, as shown in Fig. 3(a) and (b), consistent with the previous reports^17^,^18^,^19^,^20^. The most remarkable change of the bulk band structure induced by SOC is the significant VB splitting at the K point of WSe_2_ (see Fig. 3(b)), though this change is minor for MoS_2_ (Fig. 3(a)). The non-SOC band structure of the 1:1 superlattice (black solid line in Fig. 3(c)) appears to be a combination of the bulk band structures of MoS_2_ and WSe_2_. Wave function visualization (see Figs. 4 and S1) shows that the two highest VBs (VB1 and VB2) mainly originate from the VBs of WSe_2_; and the two lowest CBs (CB1 and CB2) are solely contributed by the CBs of MoS_2_. This combination significantly reduces the K-K bandgap. The VBM, a mixture of MoS_2_ and WSe_2_ states, is found at K, which is 60 meV higher in energy than the VB1 at Γ. We align the SOC corrected band structure (red dashed line in Fig. 3(c)) to the non-SOC one by VB1 at the Γ point in order to better understand the role of SOC. Similar to the WSe_2_ case, the SOC correction enlarge the gap between VB1 and VB2 and pushed the energy of VB1 higher of about 150 meV. Together with the CBM at K, we arrive at a direct bandgap of about 0.68 eV at K (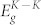). This bandgap is also tunable by in-layer and normal-layer strains which change the inter- and intra-layer electronic coupling moving upwards or downwards the energy of VB at the K point. In light of this, a superlattice could experience a direct to indirect transition under a certain external strain, as observed in Fig. 2(b) and (c).

The microscopic physics is further revealed by plotting the wave functions of CB1, CB2, VB1, and VB2 at the K point. We denote them as 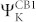, 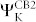, 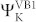, 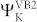, and visualize them by plotting their square norm. Figures 4(a)–(f) clearly show that 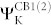 is mainly composed of Mo 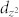 and S 3-fold *p* orbitals. Although 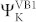 and 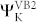 are separated in energy, Figs. 4(g)–(l) explicitly show that they originate from the same combination of inlayer 3-fold W *d* and Se *p* states. The results without SOC thus indicate that stacking the materials breaks the energetic degeneracy of VB1 and VB2 but keeps the composition of their wave functions. By including SOC, on the other hand, the composition of the wave functions are also broken as shown by panels (*b*, *c*, *e*, *f*, *h*, *i*, *k*, *l*) of Fig. 4. The wave function mainly resides at one side of a bilayer at a certain K point, for example, most portion of 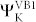 locates at the right side of the MoS_2_/WSe_2_ interface, while 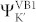 at the left side [Fig. 4(h,i)]. In other words, SOC makes the originally degenerate K and K′ points distinguishable. The enlarged separation moves the VBM from the Γ to the K point, bringing about the direct K-K gap in these superlattices. We conclude that SOC plays a critical role to produce the direct bandgaps in the proposed superlattices.

**Figure 5:**
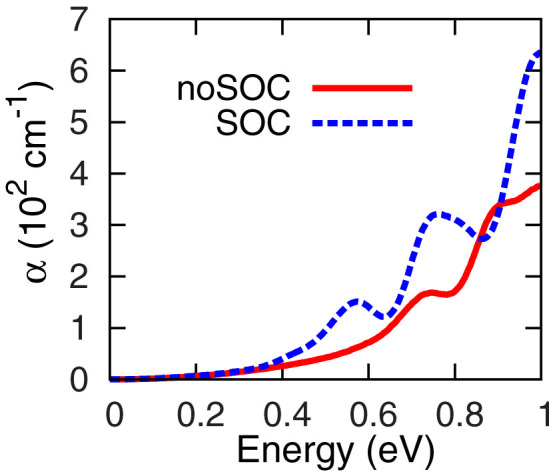
Optical absorption spectra of the 1:1 superlattice under PBE approximation. The red line shows that without SOC and the blue line shows the SOC one.

Optical absorption spectrum directly reflects the role of SOC. Figure 5 shows the absorption spectra of the 1:1 superlattice computed at the PBE level with (blue dashed line) and without (red solid line) inclusion of SOC. The absorption edge of the first peak in the red line start at about 0.5 eV, which corresponding to the fundamental bandgap of the superlattice without SOC. After turning on the SOC interaction, this peak definitely splits into two peaks. The lower energy one starts at roughly 0.35 eV, corresponding to the bandgap of the superlattice with inclusion of SOC. Moreover, by including SOC interaction, the absorption coefficients become much larger, which is consistent with the expected indirect-direct transition of bandgap at the PBE level. It is worthy to emphasize that the spatially separated valance and conduction bands is a desirable property since it should help to reduce the recombination of electron-hole pairs and thus lead to a process with a higher quantum efficiency, such as a charge transfer induced ultrafast photoelectron generation^28^.

In summary, we propose a novel MoS_2_/WSe_2_ superlattice that offer continuously tunable electronic structures and direct bandgaps which are robust against reasonable ranges of external strains. We identify that the robust direct bandgap is resulted from a strong spin-orbit coupling in WSe_2_ and the band alignment between MoS_2_ and WSe_2_. Either the MoS_2_/WSe_2_ thickness ratio or an additional in-layer external stress can continuously change the direct gap from 0.14 eV to 0.50 eV. The spatially separated valance (on WSe_2_) and conduction (on MoS_2_) bands is, from the application point of view, another attractive property of the proposed superlattice. On the fundamental side, if valance electrons are excited to the conduction bands by polarized light, the spatially separated VB and CB shall give rise to a magnetic moment normal to the MoS_2_/WSe_2_ interface. Finally, the distinguishable K and K′ points caused by SOC make these superlattices interesting in valley-electronics^29^,^30^,^31^. These properties strongly suggest that the proposed TMDC multilayer-based superlattices are highly promising for optoelectronic and photovoltaic systems in the infrared range, which may promote an new research field for TMDC. Indeed, very recently, a similar superlattice material comprised of PbSe and MoSe_2_ multilayers was successfully synthesized^32^ and SOC induced VB splitting was experimentally observed^33^.

## Methods

Density functional theory calculations were performed using the generalized gradient approximation for the exchange-correlation potential^34^, the projector augmented wave method^35^,^36^, and a plane wave basis set as implemented in the Vienna *ab-initio* simulation package^37^,^38^. The energy cutoff for plane-wave basis was set to 400 eV for all calculations. A *k*-mesh of 24 × 24 × 1 or 24 × 24 × 3, depending on different values of lattice parameter *c*, was adopted to sample the first Brillouin zone. In geometry optimization, dispersive interactions were considered by employing both a semi-empirical method at the PBE-D2^39^ level and a parameter-free van der Waals density function (vdW-DF) method^40^. The optB86b^41^ exchange was used to combine with the vdW correlation functional. The shape and volume of each superlattice were fully optimized and all atoms in it were allowed to relaxed until the residual force per atom was less than 0.01 eV/Å. Layer alignment in superlattices was chosen with the energetically most favored configuration, as reported in Ref. 15. The SOC correction, which may sufficiently influence band energies (0.16 eV and 0.4 eV for MoS_2_^11^,^42^ and WSe_2_^42^,^43^ respectively), was included for electronic bandstructure calculations. It was found that the van der Waals functionals play a major role for geometry and energetics, but a minor role in electronic structures^44^. Therefore, in addition to PBE calculation, a hybrid functional, namely Heyd-Scuseria-Ernzerhof (HSE06)^45^, has been employed to address a known issue of underestimated bandgaps by PBE in the bandstructure calculation of the 1:1 superlattice.

The optical properties were obtained from PBE results, and the k-mesh was doubled in calculating dielectric functions. Excitonic contributions were not considered in our calculations.

The total number of bands considered was set to be twice that used in the total-energy and bandstructure calculations.

## Author Contributions

Y.Z. and W.J. conceived the whole research and perform all calculations. F.Y. contributed to the SOC calculation of bulk TMDCs. J.W. and H.G. participate the explanation of results. Y.Z., J.W., H.G. and W.J. wrote the manuscript.

## Supplementary Material

Supplementary Information Supplementary Material

## Figures and Tables

**Table 1:** Fully relaxed lattice parameters for MoS_2_/WSe_2_ superlattices. 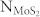 and 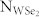 represent the number of bilayers in each superlattice for MoS_2_ and WSe_2_, respectively

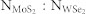	a/c PBE-D2 (vdW-DF) (Å)	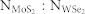	a/c PBE-D2 (vdW-DF) (Å)
1:1	3.248/25.422 (3.233/25.501)	1:1	3.248/25.422 (3.233/25.501)
1:2	3.271/38.478 (3.255/38.618)	2:1	3.229/37.919 (3.210/37.905)
1:3	3.281/51.483 (3.266/51.754)	3:1	3.220/50.326 (3.200/50.280)
1:4	3.290/64.655 (3.272/64.870)	4:1	3.219/62.477 (3.194/62.103)
1:5	3.294/77.600 (3.276/77.990)	5:1	3.214/74.932 (3.189/74.977)
