# Supplementary material for: Continuously tunable electronic structure of transition metal dichalcogenides superlattices
Source: Sci Rep. 2015 Feb 13;5:8356. doi: 10.1038/srep08356 (PMC4326700; doi:10.1038/srep08356)
Supplement: Supplementary Information — Supplementary Material [file srep08356-s1.pdf]

Supplementary Materials for the Manuscript entitled: Continuously tunable electronic structure of transition metal dichalcogenides superlattices

Yong-Hong Zhao*

College of Physics and Electronic Engineering, Institute of Solid State Physics, Sichuan Normal University, Chengdu 610068, China and Department of Physics and the Center of Theoretical and Computational Physics, The University of Hong Kong, Hong Kong, China

Feng Yang

College of Physics and Electronic Engineering, Institute of Solid State Physics, Sichuan Normal University, Chengdu 610068, China

Department of Physics, Renmin University of China, Beijing 100872, China and Beijing Key Laboratory of Optoelectronic Functional Materials & Micro-nano Devices, Renmin University of China, Beijing 100872, China

Jian Wang

Department of Physics and the Center of Theoretical and Computational Physics, The University of Hong Kong, Hong Kong, China

Hong Guo

Centre for the Physics of Materials and Department of Physics, McGill University, 3600 rue University, Montreal PQ, Canada H3A 2T8

Wei Ji†

Department of Physics, Renmin University of China, Beijing 100872, China and Beijing Key Laboratory of Optoelectronic Functional Materials & Micro-nano Devices, Renmin University of China, Beijing 100872, China

In this Supplemental Material, we present supporting information for two issues of the main text. The first is the visualized wavefunctions of the 1:1 superlattice at the center of the Brillouin zone (BZ). The second concerns band structures of the superlattices with thickness ratios of 1:5 to 5:1, and the evolution of band structures with in-layer and normal-layer strains.

Wavefunction of 1:1 superlattice

It can be seen from Fig. S1(a) that the lowest conduction band at center of BZ is composed of hybrid orbits of in-layer Mo d and S p states, analogous to VB at the K point. The valence bands ((b)-(d)) are composed of d_{z^2} states of transition metal atoms and p states of chalcogen atoms, which is similar to CB at the K point. The highest valence band (VB1) is attributed mainly to WSe_2 in combination with some MoS_2 states. For the second valence band (VB2), WSe_2 and MoS_2 contribute almost the same portion. The third valence band (VB3) is mainly composed of the MoS_2 states with some contributions from the WSe_2 states. This situation is different from that at the K point where most of the VBs are attributed to the WSe_2 states. Also due to the highest symmetry of the center of BZ, SOC has little effects on these wavefunctions, different from those at the K point where distinguishable states at K and K' are found.

Band structures of the superlattice

The calculated band structures of superlattices with MoS_2:WSe_2 ratio from 1:5 to 5:1 are shown in Fig. S2(a). The evolution of band structures of the 5:1 superlattice with in-layer and normal-layer strains are presented in Fig. S2(b) and (c). All the band structures are aligned with the highest VB at the K point. From Fig. S2(a) we observe that increasing the relative thickness of MoS_2 pushes up CBs at the K point and pulls down VBs at the Γ point, thereby causing an increase of the band gap as well as a change from indirect (Γ-K) to direct (K-K) band gap.

Fig. S2(b) shows the band structures of the 5:1 superlattice as a function of in-layer strains. The in-layer stretching strain pulls down CBs at K and pushes up VBs at Γ thus both indirect and direct band gaps are reduced. This causes a transition from direct to indirect band gap at about 2% stretching and eventually the gap becomes zero at stretching ratio of about 4%. The in-layer compressing strain significantly lifts up CBs at K and brings down CBs at some intermediate point (I) between Γ and K; it also brings down VBs at Γ. As a result, the compressing strain eventually leads to an indirect band

gap between K and I.

Fig. S2(c) shows the band structures of 5:1 superlattice as a function of the normal-layer compressing strain. The most noticeable effect is the lifting-up of VBs only at the Γ point. There is a transition from direct to indirect band gap at a normal-layer compression ratio of about 4.3%, corresponding to an external pressure of about 3GPa.

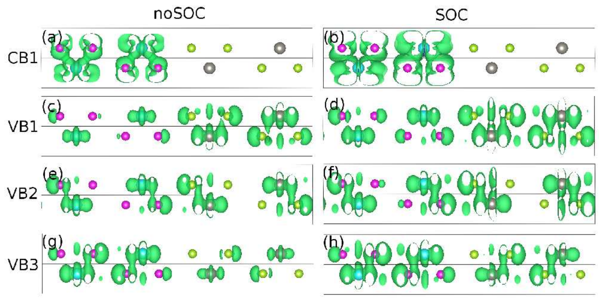

FIG. 1. (Color online) Visualized wavefunction of the 1:1 superlattice at the center of the Brillouin zone. (a) For the lowest conduction band; (b)-(d) for the highest three valence bands whose energies decrease from VB1 to VB3. The upper panels are for that without SOC while the lower panels with SOC. Isosurface is 0.001.

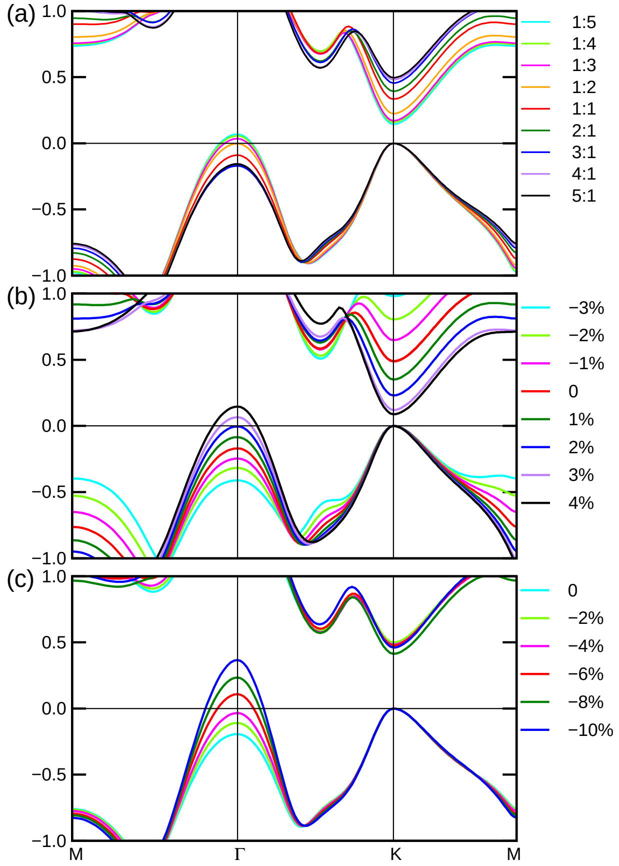

FIG. 2. (Color online) Band structure for (a) superlattice with the MoS_2:WSe_2 ratio from 1:5 to 5:1, corresponding to Fig. 4(a). (b) 5:1 superlattice with in-layer strain from -3% to 4% comparing to Fig. 4(b). (c) 5:1 superlattice with normal-layer compressing strain up to 10% comparing to Fig. 4(c).
